# The risk of radiation necrosis from combined radiotherapy and BRAF inhibitor in lung adenocarcinoma brain metastases: a comprehensive review and future perspectives

**DOI:** 10.3389/fonc.2026.1622947

**Published:** 2026-02-06

**Authors:** Lina Yang, Yanan He, Yong Peng, Mao Sun, Zheng Tang, Liang Du, Yongzhong Wu, Wei Zhou, Dingyi Yang

**Affiliations:** 1 Department of Radiation Oncology, Chongqing University Cancer Hospital, Chongqing, China; 2 Chongqing Key Laboratory of Molecular Oncology and Epigenetics, The First Affiliated Hospital of Chongqing Medical University, Chongqing, China; 3 School of Pharmaceutical Science, Wuhan University, Wuhan, China; 4 People’s Second Hospital of Dazu District, Chongqing, China; 5 Chongqing Key Laboratory of Translational Research for Cancer Metastasis and Individualized Treatment, Chongqing University Cancer Hospital, Chongqing, China

**Keywords:** BRAF-V600E mutation, brain metastases, cranial radiotherapy complications, non-small-cell lung cancer, radiation necrosis

## Abstract

The management of lung adenocarcinoma with brain metastases (BMs) is particularly challenging when BRAF-V600E mutations emerge as a resistance mechanism to EGFR tyrosine kinase inhibitors. While the combination of BRAF/MEK inhibitors (e.g., dabrafenib and trametinib) and radiotherapy (RT) is a pivotal therapeutic strategy, it significantly increases the risk of radiation necrosis (RN). This review summarizes the current understanding of the molecular mechanisms and risk factors underlying RN development in this specific patient population. We detail how BRAFi exacerbate RT-induced vascular injury, blood-brain barrier (BBB) disruption, and inflammatory responses, focusing on MAPK pathway modulation, VEGF signaling inhibition, and paradoxical pathway activation. Clinical correlations regarding treatment timing and regimen choice are discussed. Finally, we propose comprehensive strategies to mitigate RN risk, including optimized treatment sequencing, RT dose adjustments, advanced imaging for early detection, and novel approaches for vascular repair. This review underscores prospective studies and standardized guidelines are urgently needed to refine combination strategies and improve outcomes for these patients.

## 1 Introduction

Lung adenocarcinoma, the predominant subtype of non-small cell lung cancer (NSCLC), is frequently driven by actionable oncogenic mutations. While EGFR mutations are effectively targeted by tyrosine kinase inhibitors (TKIs) such as osimertinib, acquired resistance often develops. The BRAF-V600E mutation has emerged as a key resistance mechanism, shifting the treatment paradigm to alternative targeted therapies (TTs), including dabrafenib and trametinib (1, 2). Brain metastases (BMs) develop in approximately 20-40% of advanced NSCLC patients and are a leading cause of morbidity and mortality, with a median overall survival of less than one year (3, 4). Local radiotherapy (RT), including stereotactic radiosurgery (SRS) and whole-brain radiotherapy (WBRT), remains a cornerstone of BMs management, providing durable local control (5–7). Combining RT with targeted agents has shown promise for improving intracranial control, yet the optimal integration of these modalities remains undefined, particularly for BRAF inhibitors (8, 9).

Complicating this approach is the risk of RN, a serious complication of cranial irradiation characterized by neuroinflammation, blood-brain barrier disruption, and cognitive decline (10–13). The combination of BRAF inhibitors (BRAFi) with radiotherapy (RT) has shown efficacy in controlling BRAF-mutant tumors but is associated with an increased risk of severe radiation necrosis, particularly in the central nervous system (CNS) (14). When BRAFi (especially first-generation inhibitors like vemurafenib) are administered concurrently with RT, they may synergistically disrupt critical pro-survival and repair pathways in the vascular endothelium. Furthermore, the “paradoxical activation” of the MAPK pathway by these inhibitors in cells with wild-type BRAF (such as normal endothelial cells in the irradiated field) may create a pro-inflammatory milieu, enhancing vascular leakage and inflammatory cell recruitment. This combined insult amplifies the cascade of endothelial dysfunction, abnormal angiogenesis, and eventual vessel obliteration, thereby significantly increasing the probability of developing severe, symptomatic RN (15, 16).

This review synthesizes current evidence on the mechanisms and risk factors for RN in patients with BRAF-mutant lung adenocarcinoma receiving combined BRAFi and RT. We highlight the urgent need for multidisciplinary management strategies and future research to optimize therapeutic outcomes while mitigating this debilitating toxicity.

## 2 BRAF inhibitors: classification and mechanism of action

BRAFi are a class of targeted anticancer drugs designed to block the activity of the BRAF kinase, a key component of the MAPK/ERK signaling pathway. This pathway is frequently dysregulated in cancers, most commonly via the BRAF V600E mutation. BRAFi specifically bind to the ATP-binding site of the mutant BRAF kinase, inhibiting its constitutive activity and downstream signaling, leading to cell cycle arrest and apoptosis (17).

It is crucial to distinguish their mode of action regarding wild-type BRAF (BRAF WT). First-generation BRAFi (e.g., vemurafenib, dabrafenib) are considered selective or mutant-selective inhibitors. They potently inhibit monomeric mutant BRAF (especially V600E). However, in cells with active upstream signaling (e.g., RAS activation), they can paradoxically activate the MAPK pathway by promoting dimerization of BRAF WT with CRAF, a phenomenon known as “paradoxical activation” (18). Second-generation “paradox breaker” inhibitors (e.g., PLX8394) are designed to inhibit both mutant and wild-type BRAF without inducing this paradoxical activation (19). Pan-RAF inhibitors (e.g., sorafenib, LY3009120) target all RAF isoforms (ARAF, BRAF, CRAF) regardless of mutation status (20, 21). **Table 1** lists the current available BRAFi in market.

**Table 1:** Commonly available BRAF inhibitors.

Inhibitor (Trade name)	Generic name	Primary target specificity	Key approved indication(s)	Notable clinical trial (e.g.)
Zelboraf	Vemurafenib	Selective for BRAF V600 mutant	Unresectable or metastatic melanoma with BRAF V600E mutation	BRIM-3 (NCT01006980)
Tafinlar	Dabrafenib	Selective for BRAF V600 mutant	Melanoma, NSCLC, ATC with BRAF V600E mutation	BREAK-3 (NCT01227889)
Braftovi	Encorafenib	Selective for BRAF V600 mutant	Used with binimetinib/cetuximab for BRAF-mutant melanoma/CRC	COLUMBUS; BEACON CRC (NCT02928224)

## 3 Overview of radiation-induced tissue injury, radiation necrosis, and vascular remodeling

1. Radiation-Induced Tissue Injury (RITI) is a broader term encompassing the pathological changes in normal tissues following radiotherapy. It is a dynamic process involving acute inflammation and chronic, progressive fibrosis. The pathogenesis involves DNA damage in parenchymal and vascular endothelial cells, triggering a persistent pro-inflammatory and pro-fibrotic cytokine cascade (22).
2. Radiation necrosis(RN) is a delayed, non-malignant complication of CNS by RT, typically occurring 3 months to several years post-treatment, characterized by focal coagulative necrosis of the white matter. It results from a combination of direct glial cell damage and, crucially, vascular endothelial injury, leading to increased blood-brain barrier(BBB) permeability, edema, and thrombosis (23).

RN primarily driven by vascular dysfunction, immune-mediated inflammation, and oxidative stress, all contributing to disruption of the BBB, ischemic injury, and chronic neuroinflammation (24). In which, initiated primarily by radiotherapy-induced disruption of BBB and direct neural cell damage. Ionizing radiation causes structural and functional damage to the BBB by inducing endothelial cell apoptosis, tight junction protein degradation, and increased vascular permeability, leading to leakage of plasma components and infiltration of inflammatory cells into the brain parenchyma (25). This BBB disruption facilitates neuroinflammation and oxidative stress, which are pivotal in the pathogenesis of RN (24). Key molecular pathways involved include VEGF-mediated endothelial damage, cGAS-STING-driven innate immune activation, and radiation-induced mitochondrial dysfunction (13).

Microglial activation is a hallmark of RN, with these resident immune cells releasing pro-inflammatory cytokines such as IL-6, TNF-α, and reactive oxygen species (ROS), exacerbating neuronal injury and perpetuating a vicious cycle of inflammation. Oxidative stress, resulting from excessive ROS production and impaired antioxidant defenses, further damages lipids, proteins, and nucleic acids within neural cells, contributing to cellular dysfunction and death (26, 27).

Several factors influence the risk of RN, including radiation dose, fractionation schedule, and treatment volume. Higher biologically effective doses (BED), larger treatment fields, and retreatment with cumulative doses exceeding 100 Gy increase RN risk (28). Additionally, tumor biology (e.g., BRAF mutations, VEGF overexpression) and patient-specific factors (e.g., age, pre-existing vascular disease) may further predispose individuals to RN (29).

(3) Radiation directly damages vascular endothelial cells, inducing senescence, apoptosis, and increased expression of adhesion molecules (e.g., ICAM-1, VCAM-1). This initiates a chronic state of vascular inflammation and dysfunction. Key alterations include: Loss of nitric oxide (NO) bioavailability, increased oxidative stress, and upregulation of pro-inflammatory cytokines (e.g., TNF-α, IL-1β, IFN-γ). Disruption of tight junctions and the basement membrane increases permeability. Radiation promotes the expression of VEGF and other angiogenic factors as a compensatory response. However, the newly formed vessels are often abnormal—dilated, tortuous, and leaky—with poor pericyte coverage, failing to restore proper perfusion. Chronic inflammation leads to vessel wall thickening, hyalinization, and thrombosis, culminating in reduced vascular density and tissue ischemia. The persistent release of TGF-β drives the differentiation of fibroblasts into myofibroblasts, resulting in perivascular and parenchymal fibrosis, which further compromises tissue architecture and function (30, 31).

## 4 Clinical evidence of RN risk with BRAF inhibitors and RT

The combination of RT with molecular targeted therapies (TTs) represents a cornerstone of modern oncology, offering potent synergy for tumor control. However, this synergy can also amplify off-target toxicity in normal tissues. In the context of brain metastases, concurrent RT and BRAFi has been increasingly associated with an elevated risk of RN (29, 32).

The clinical evidence supporting this association, however, is unevenly distributed across cancer types. As summarized in **Table 2**, the most compelling data originate from studies in melanoma and glioblastoma, where the use of BRAFi is well-established. These studies consistently report a higher incidence and sometimes atypical presentation of RN following SRS or fractionated RT when combined with targeted agents. For instance, a illustrative case involved a patient with MAP2K1-mutant melanoma who developed RN nine months post-SRS while on trametinib and low-dose dabrafenib, which was subsequently managed with bevacizumab (33). This and other cases underscore the clinical reality of this interaction.

**Table 2:** Summary of clinical evidence on BRAF inhibitors and radiotherapy-associated necrosis.

Study	Study year	Study type	N (patients)	Median dose (Gy)	Radiotherapy	Targeted therapy	Toxicity incidence/description	Start of targeted therapy	Tumor type	Subgroup analysis
Kim et al. (12)	2017	Retrospective cohort study	218 (concurrent TTs)	24 (18–24)	SRS± WBRT	VEGFR, EGFR, ALK, BRAF inhibitors	Brain necrosis: 8.8–15.6%; VEGFR TKIs increased RN risk (13% vs. 5.3%); EGFR TKIs elevated RN risk (14% vs. 5.3%). BRAF-i showed no significant increase in RN.	Concurrent	BMs (NSCLC, renal, melanoma, and breast)	EGFR TKIs with SRS+WBRT significantly elevated RN risk (20% vs. 3.3%). Peaking 4.5–9 months post-SRS with concurrent therapies
Kroeze et al. (13)	2017	Systematic Review	644 (cranial SRT)	18–27	SRT	BRAF, EGFR, ALK inhibitors	BRAFi: Grade 3/4 cerebral oedema in 15%; intratumoral hemorrhage in up to 23%.	Concurrent	BMs, melanoma, NSCLC	Higher risk of severe CNS toxicity with BRAF-i. But EGFR-i show good tolerability with cranial SRT.
Pierre-Yves et al. (28)	2021	Systematic Review	327 patients	24 (6–24), 21–24, 25	SRT	Trastuzumab and T-DM1, EGFR, BRAF, MEK, ALK inhibitors	No significant increase in RN or clinical toxicity	Concurrent or at the time of SRT (± 30 days)	BMs (melanoma, lung, breast, renal carcinoma)	Highlighted safety of concurrent SRT with TTs, with no clinically significant increase in RN
Maikel et al. (29)	2017	Literature Review	Multiple studies	20–30 (WBRT); 15–20 (SRS)	WBRT, SRS	EGFR, BRAFi, Sunitinib, Sorafenib	Erlotinib, gefitinib and vemurafenib showed increased neurotoxicity risks (fatigue, brain hemorrhage).	Concurrent	BMs	Most TTs don’t increase neurotoxicity with CRT. But sunitinib and Sorafenib may act as radiosensitizers.

In stark contrast, direct clinical evidence specifically linking BRAFi to RN in NSCLC remains scarce. The limited number of studies, as cataloged in **Table 2**, highlights a significant knowledge gap. This paucity of data is particularly concerning given the unique pathophysiology of lung cancer brain metastases and the increasing adoption of these targeted therapies in the NSCLC setting. The mechanisms underlying this increased risk—potentially involving radiosensitization, vascular toxicity, inflammatory and tissue repair interference—are explored in the following section.

## 5 Synergistic mechanisms of BRAFi and RT in inducing radiation necrosis

BRAFi, used particularly in BRAF-mutant melanoma and lung adenocarcinoma, may potentiate radiation-induced neurotoxicity through mechanisms that are not fully elucidated but likely involve exacerbation of oxidative stress and inflammatory responses (16, 32). BRAFi such as dabrafenib and MEK inhibitors such as trametinib target the MAPK/ERK signaling pathway, suppressing tumor proliferation. When combined with radiation, BRAFi may amplify additional toxicities by enhancing radiosensitivity through several mechanisms:

### 5.1 Radiosensitization via MAPK pathway modulation

The combination of BRAFi with RT leads to complex, cell-context-dependent interactions within the MAPK/ERK pathway that ultimately enhance radiation toxicity. In BRAF V600E-mutant tumor cells, BRAFi potently inhibit constitutive MAPK signaling. This inhibition not only suppresses proliferation but also critically impairs the cellular response to radiation-induced DNA damage. Specifically, BRAFi abrogate the radiation-induced G2/M cell-cycle checkpoint arrest, a vital period for DNA repair, forcing cells with unrepaired double-strand breaks into mitosis, leading to mitotic catastrophe and enhanced cell death. This constitutes a primary mechanism of radiosensitization in the tumor compartment (34).

Paradoxically, in BRAF wild-type (WT) cells—such as vascular endothelial cells, normal glial cells, and infiltrating immune cells within the tumor microenvironment—first-generation BRAFi can induce hyperactivation of the MAPK pathway. This occurs through drug-induced promotion of RAF dimerization (e.g., BRAF WT-CRAF), leading to feedback-driven, ligand-dependent ERK signaling. In the context of an irradiated field, this “paradoxical activation” in normal tissues exacerbates radiation-induced stress, promoting excessive inflammatory cytokine release, endothelial dysfunction, and cytotoxic effects on supportive glial cells. This differential effect—inhibition in tumor cells versus potential activation in surrounding normal tissue—creates a dichotomy that underlies both the therapeutic efficacy and the heightened risk of normal tissue complications like radiation necrosis (18, 35).

**Figure 1:**
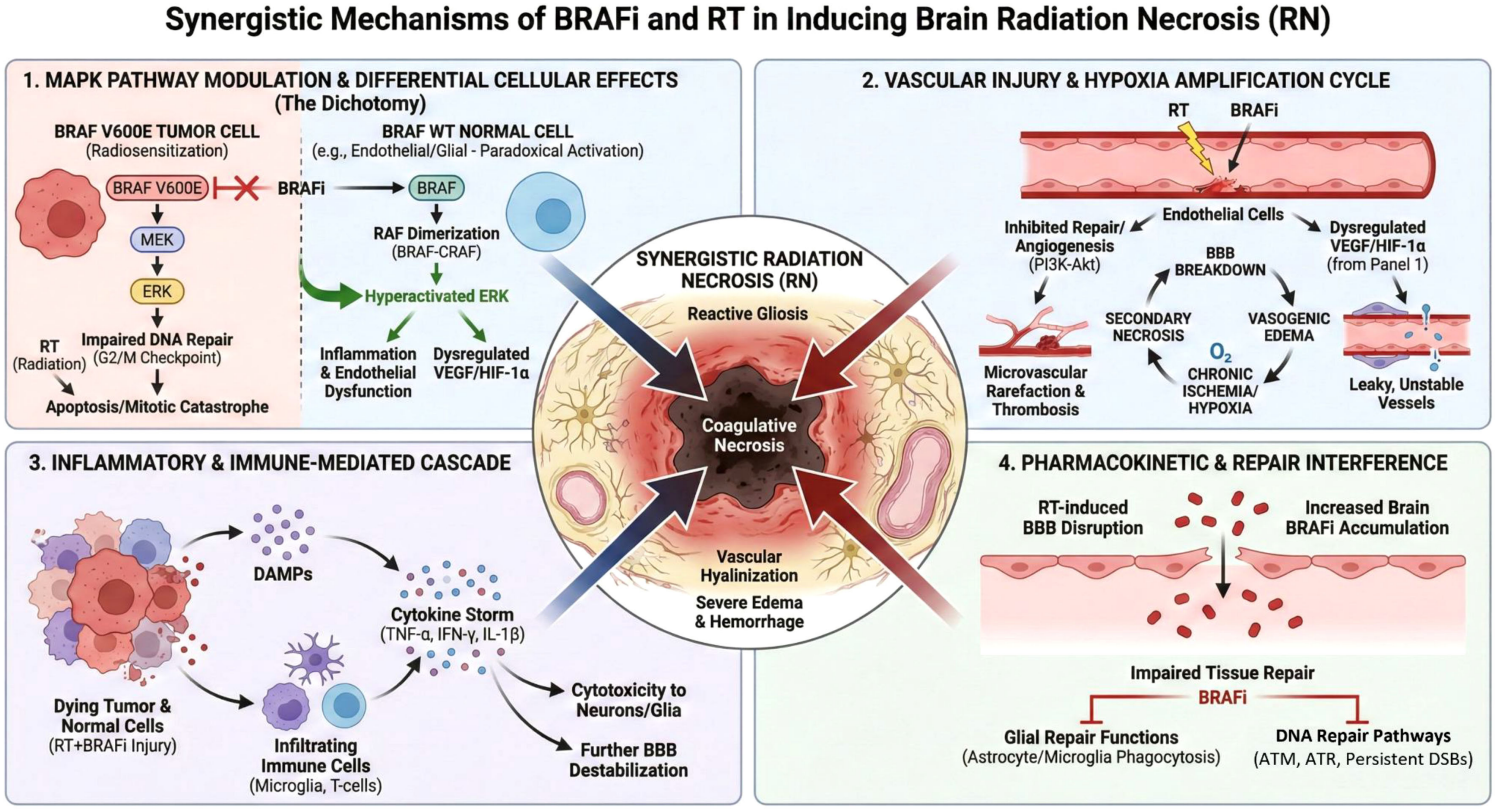
Synergistic mechanisms of BRAFi and RT in inducing radiation necrosis.

The phenomenon of “paradoxical activation” is central to the vascular toxicity of combined therapy. In normal cells with intact RAS signaling, BRAFi binding to one protomer of a RAF dimer (e.g., BRAF) allosterically promotes transactivation of the drug-free partner (e.g., CRAF). This leads to hyperactivation of the MEK-ERK pathway (17). In the irradiated tissue, which is already hypoxic and rich in growth factors, this paradoxically activated ERK signaling further stabilizes HIF-1α and increases VEGF production. However, this VEGF surge is pathologic. It promotes the formation of leaky, malformed, and unstable vessels that lack proper pericyte coverage and are incapable of adequate perfusion (36). The MAPK pathway is integral to VEGF-mediated angiogenic signaling, its inhibition by BRAFi disrupts the compensatory angiogenic response necessary for recovery from RT-induced damage (37). **Figure 1** illustrates the possible synergistic mechanism of the combined use of BRAFi and RT in inducing radiation necrosis.

### 5.2 Vascular injury and hypoxia amplification

The synergistic vascular toxicity of BRAFi and RT is a cornerstone in the pathogenesis of severe RN. RT directly damages vascular endothelial cells, initiating a cascade of senescence, apoptosis, and increased permeability. BRAFi potentiate this injury by inhibiting endothelial cell proliferation, migration, and survival signals that are crucial for vascular repair and maintenance. The combined insult leads to microvascular rarefaction, characterized by a loss of functional capillaries, and persistent BBB breakdown.

BRAFi compromise endothelial repair, leading to increased vascular permeability, microvascular thrombosis, and sustained edema (16). VEGF upregulation, exacerbated by RT, promotes capillary leakage and inflammatory infiltration, further weakening the BBB and exacerbating radiation-induced injury (38). The failure to regenerate a competent vascular network results in chronic ischemia and hypoxia within the irradiated tissue bed. This hypoxic environment, in turn, promotes secondary necrosis, exacerbates edema, and creates a vicious cycle that drives the expansion of necrotic lesions. Histopathologically, this is evident as fibrinoid necrosis, vessel wall hyalinization, and thrombosis.

BRAFi increase ROS production and inhibit NRF2-mediated antioxidant defense, leading to prolonged oxidative stress and cellular injury. Radiation exposure already generates ROS, and the combined effect may amplify oxidative damage in both tumor and normal brain tissue (39, 40).

### 5.3 Inflammatory and immune-mediated mechanisms

The convergence of BRAFi and RT potently activates and sustains a pro-inflammatory milieu that amplifies tissue damage. BRAFi enhance antigen presentation and increase the release of damage-associated molecular patterns (DAMPs) from dying tumor cells. Concurrently, RT induces a robust sterile inflammatory response by releasing DAMPs from both tumor and injured normal cells. The combination leads to an amplified recruitment and activation of innate immune cells (e.g., microglia, macrophages) and adaptive immune cells (e.g., T-cells) into the tumor microenvironment and surrounding brain parenchyma (34, 41).

This infiltrate releases high levels of pro-inflammatory cytokines such as TNF-α, IFN-γ, and IL-1β. These cytokines further destabilize the BBB, promote vasogenic edema, and exert direct cytotoxic effects on oligodendrocytes and neurons. The resulting histopathology is a blend of coagulative necrosis, intense perivascular and parenchymal lymphocytic inflammation, and reactive gliosis—features that reflect the compounded effect of both treatments and distinguish this form of necrosis from that caused by RT alone.

### 5.4 Tissue repair and pharmacokinetic interference

Emerging evidence suggests that BRAFi may interfere with the intrinsic repair capacity of the CNS beyond direct vascular toxicity (16). Astrocytes and microglia, essential for maintaining homeostasis and coordinating repair after injury, rely on finely tuned intracellular signaling. BRAFi exposure can impair the protective and reparative functions of these glial cells, potentially by disrupting MAPK-dependent trophic support and phagocytic activity. This impairment slows the clearance of cellular debris and the resolution of inflammation, prolonging the injury phase (17, 30).

Furthermore, the RT-induced disruption of the BBB may alter the pharmacokinetics of BRAFi. The compromised barrier can lead to increased and sustained local drug accumulation in the irradiated brain tissue, resulting in higher-than-expected exposure and prolonged on-target (and off-target) effects on both tumor and normal cells. This localized pharmacokinetic shift creates a focus of intensified pharmacodynamic activity, concentrating the mechanisms described above (vascular injury, paradoxical signaling, inflammation) precisely within the region most vulnerable to necrosis, thereby explaining the focal and severe nature of this complication (16, 39, 42).

## 6 Mechanistic interplay between BRAFi and VEGF signaling in the context of RT

### 6.1 Direct suppression of tumor VEGF signaling

In BRAF V600E-mutant tumor cells, the constitutively active BRAF-MEK-ERK axis drives oncogenic gene expression programs, including the stabilization of Hypoxia-Inducible Factor 1-alpha (HIF-1α) and the transcription of pro-angiogenic factors like VEGF-A. BRAFi directly block this pathway, leading to decreased ERK-mediated phosphorylation events, reduced HIF-1α protein stabilization, and subsequent downregulation of VEGF transcription and secretion (15). This anti-angiogenic effect is therapeutically beneficial, as it reduces tumor neovascularization, normalizes the chaotic tumor vasculature, and can improve drug delivery. However, when combined with radiotherapy (RT), this systemic suppression of pro-repair VEGF signaling can be detrimental. In normal tissues within the radiation field, the impaired angiogenic response prevents the necessary vascular repair after RT-induced injury, exacerbating ischemia and culminating in necrosis (30).

### 6.2 Dual and paradoxical effects on VEGF signaling

The impact of BRAFi on VEGF signaling is profoundly context-dependent, creating a dichotomy between tumor and normal tissue. While BRAFi suppress the pathway in mutant cells, they can paradoxically enhance MAPK and VEGF signaling in BRAF WT cells, such as endothelial cells, fibroblasts, and astrocytes (17). This aberrant activation can lead to increased HIF-1α activity and VEGF secretion in the stromal compartment, resulting in a dysregulated, non-productive angiogenic stimulus.

### 6.3 Impaired angiogenic repair post-radiotherapy

RT inflicts direct damage on the microvasculature, causing endothelial cell apoptosis and capillary rupture. The natural tissue response to this injury is a compensatory upregulation of VEGF-driven angiogenesis to restore perfusion and initiate repair. BRAFi disrupt this critical recovery process. By suppressing VEGF signaling, they impair endothelial cell proliferation, migration, and survival, effectively blocking the regeneration of a functional vascular network (41). What would otherwise be a transient, repairable injury becomes a state of chronic ischemia, hypoxia, and ultimately, progressive necrosis.

## 7 Clinical risk factors, manifestations and diagnosis of RN

Clinical data, primarily from melanoma, confirm the heightened RN risk with BRAFi+RT combination. Key risk factors include: Treatment Timing: Concurrent or closely sequenced administration (within ~3 days) carries the highest risk (16). RT Modality and Dose: SRS, with its high dose per fraction, is associated with a greater risk of symptomatic RN compared to fractionated WBRT when combined with BRAFi, likely due to more intense focal vascular injury (43).

RN manifests clinically in distinct phases with characteristic symptoms and imaging features that evolve over time. In the acute phase, patients commonly present with symptoms such as headache, cognitive dysfunction, and neurological deficits, reflecting early inflammatory and edema processes in the brain tissue. These acute symptoms may be transient but are indicative of initial radiation-induced cellular and vascular stress. As the injury progresses to the delayed or late phase, more chronic manifestations emerge, including white matter abnormalities, brain atrophy, and persistent cognitive decline. These late effects correspond to irreversible structural damage such as demyelination, necrosis, and microvascular injury (44, 45).

Advanced neuroimaging techniques, such as magnetic resonance imaging (MRI), in which including conventional T2-weighted imaging and diffusion tensor imaging (DTI), reveals white matter changes, edema, and atrophic changes, play a pivotal role in the diagnosis and monitoring of RN. But, distinguishing RN from tumor progression is challenging, particularly in patients on targeted therapy, as both conditions present with contrast-enhancing lesions and perilesional edema on MRI. Advanced neuroimaging techniques, such as MR spectroscopy, perfusion MRI, and PET-CT, can improve diagnostic specificity by evaluating metabolic and vascular changes (46). Additionally, routine molecular profiling may help identify high-risk patients early, facilitating preemptive intervention.

The combination of clinical symptomatology and multimodal imaging enhances the accuracy of RN diagnosis, enabling differentiation from tumor progression and guiding therapeutic decisions. However, challenges remain in correlating imaging findings with clinical symptoms due to the complex pathophysiology and heterogeneous presentation of RN. Rodent models have provided insights into cellular and molecular mechanisms but do not fully replicate human clinical and radiological features, underscoring the need for further translational research to optimize diagnostic criteria and imaging biomarkers for RN. Given these complexities, multidisciplinary collaboration involving oncologists, radiation oncologists, and neurologists is essential for optimizing diagnosis and management.

## 8 Strategies to mitigate combined toxicity and enhance vascular repair

### 8.1 Treatment timing and sequencing

A strategic treatment hiatus is the most widely recommended mitigation strategy. Holding the BRAFi for 3–5 days before and after each RT fraction, particularly SRS, allows a window for the initiation of endogenous vascular repair mechanisms without the inhibitory pressure of the drug, significantly reducing the incidence of severe necrosis (47).

### 8.2 Rational drug regimen choice

Combining a BRAFi with a MEK inhibitor (MEKi) is a standard-of-care approach that not only improves antitumor efficacy but also reduces the skin and normal tissue toxicity associated with paradoxical MAPK activation. The MEKi blunts the ERK activation downstream of paradoxical RAF dimerization, thereby mitigating this key driver of pathologic signaling in normal tissues (48).

### 8.3 Optimized radiotherapy design

Employing hypofractionated RT schedules instead of single-fraction SRS can distribute the vascular injury over time, allowing for repair between fractions. Furthermore, employing highly conformal techniques and adhering to strict normal tissue dose constraints (e.g., for brainstem or hippocampi) can minimize the volume of sensitive tissue exposed to high doses.

### 8.4 Active management of established necrosis

Clinicians must be prepared to manage necrosis aggressively. High-dose corticosteroids are first-line to reduce edema. For steroid-refractory cases, bevacizumab (a VEGF-neutralizing antibody) has shown efficacy in numerous studies. By neutralizing the pathologic, leaky-vessel-inducing VEGF, it can reduce edema and improve radiographic appearance, though its use remains off-label (49).

### 8.5 Vigilant monitoring and adaptive treatment

Early detection is key. Frequent surveillance MRI with advanced sequences (e.g., perfusion-weighted imaging) is crucial to distinguish necrosis from progression. A low threshold for neurologic assessment and prompt adjustment of systemic therapy upon suspicion of necrosis can prevent irreversible neurological deficits.

### 8.6 Investigational pro-angiogenic and pro-repair strategies

Paradoxically, after the acute anti-cancer treatment phase, strategies to promote healthy angiogenesis may be beneficial for repairing RT-induced damage. The secretome of mesenchymal stem cells (MSCs) is rich in pro-angiogenic, anti-inflammatory, and trophic factors that can promote the formation of stable vasculature and mitigate inflammation. MSC-derived exosomes are under investigation as a cell-free therapy to stimulate vascular regeneration and repair long-term tissue damage after oncologic therapy (50).

## 9 Conclusion and future perspectives

In conclusion, the combination of BRAFi and RT for BRAF-mutant lung cancer brain metastases is a potent but double-edged sword, offering improved tumor control at the cost of increased RN risk. This review underscores the need for strategic BRAFi -RT integration, personalized dose adaptation, and vigilant monitoring. This interplay between RN and BRAFi effects necessitates a comprehensive understanding of molecular and cellular dynamics to inform clinical decision-making. Clinically, optimizing treatment regimens requires meticulous integration of molecular tumor profiling with advanced radiotherapeutic techniques. Tailoring the timing, dosage, and sequencing of targeted agents alongside precision radiotherapy modalities such as stereotactic radiosurgery or intensity-modulated radiotherapy can mitigate the risk of RN. Moreover, early pharmacologic interventions aimed at neuroprotection should be explored and incorporated into therapeutic protocols to safeguard neural tissue without compromising oncologic efficacy.

Looking forward, future research must prioritize elucidating the intricate molecular mechanisms underpinning RN, particularly the interactions between targeted therapies and radiation at the cellular and microenvironmental levels. Additionally, clinical trials evaluating innovative combination strategies that balance tumor control with neurotoxicity prevention are imperative. This review highlights the critical need for continued translational research and clinical innovation to achieve this balance and improve the prognosis for this challenging patient cohort.

## References

[1] Nana FA Ocak S . Targeting BRAF activation as acquired resistance mechanism to EGFR tyrosine kinase inhibitors in EGFR-mutant non-small-cell lung cancer. Pharmaceutics. (2021) 13(9):1478. doi: 10.3390/pharmaceutics13091478, PMID: 34575554 PMC8471192

[2] Liu D Ding K Yin K Peng Z Li X Pan Y . A real world analysis of secondary BRAF variations after targeted therapy resistance in driver gene positive NSCLC. Sci Rep. (2024) 14:20302. doi: 10.1038/s41598-024-71143-6, PMID: 39218919 PMC11366755

[3] Sayin SI Eklund EA Ali KX Dzanan JJ Xylander M Dankis M . Distinct metastatic organotropism shapes prognosis in lung adenocarcinoma with brain metastasis. Front Oncol. (2025) 15:1569517. doi: 10.3389/fonc.2025.1569517, PMID: 40291914 PMC12031661

[4] Souza VGP Telkar N Lam WL Reis PP . Comprehensive analysis of lung adenocarcinoma and brain metastasis through integrated single-cell transcriptomics. Int J Mol Sci. (2024) 25:24. doi: 10.3390/ijms25073779, PMID: 38612588 PMC11012108

[5] Mehta GU Raza SM . Management of skull base metastases. Neurosurg Clin N Am. (2020) 31:659–66. doi: 10.1016/j.nec.2020.06.013, PMID: 32921360

[6] Gondi V Bauman G Bradfield L Burri SH Cabrera AR Cunningham DA . Radiation therapy for brain metastases: an ASTRO clinical practice guideline. Pract Radiat Oncol. (2022) 12:265–82. doi: 10.1016/j.prro.2022.02.003, PMID: 35534352

[7] El Shafie RA Böhm K Weber D Lang K Schlaich F Adeberg S . Palliative radiotherapy for leptomeningeal carcinomatosis-analysis of outcome, prognostic factors, and symptom response. Front Oncol. (2019) 8:641. doi: 10.3389/fonc.2018.00641, PMID: 30671384 PMC6331444

[8] Deng G Zhang Q Fan J Zhao C Jiao H Li Z . Optimal intervention timing for craniocerebral radiotherapy in EGFR mutant lung adenocarcinoma patients with brain metastases. BMC Cancer. (2024) 24:1571. doi: 10.1186/s12885-024-13363-7, PMID: 39716108 PMC11664826

[9] Swalduz A Beau-Faller M Planchard D Mazieres J Bayle-Bleuez S Debieuvre D . Real-world efficacy of the dabrafenib-trametinib (D-T) combination in BRAF V600E-mutated metastatic non-small cell lung cancer (NSCLC): Results from the IFCT-2004 BLaDE cohort. Lung Cancer. (2024) 199:108038. doi: 10.1016/j.lungcan.2024.108038, PMID: 39616778

[10] Rubino S Oliver DE Tran ND Vogelbaum MA Forsyth PA Yu HM . Improving brain metastases outcomes through therapeutic synergy between stereotactic radiosurgery and targeted cancer therapies. Front Oncol. (2022) 12:854402. doi: 10.3389/fonc.2022.854402, PMID: 35311078 PMC8924127

[11] Ye C Handa P Sahgal A Lo S Vellayappan B . Risk-reduction strategies for late complications arising from brain metastases treated with radiotherapy: a narrative review. Chin Clin Oncol. (2022) 11:13. doi: 10.21037/cco-21-121, PMID: 35400164

[12] Kim JM Miller JA Kotecha R Miller JA Kotecha R Xiao R Juloori A Ward MC . The risk of radiation necrosis following stereotactic radiosurgery with concurrent systemic therapies. Int J Radiat OncologyBiologyPhys. (2017) 99:S159–. doi: 10.1016/j.ijrobp.2017.06.367, PMID: 28434110

[13] Kroeze SGC Fritz C Hoyer M Lo SS Ricardi U Sahgal A . Toxicity of concurrent stereotactic radiotherapy and targeted therapy or immunotherapy: A systematic review. Radiother Oncol. (2017) 53:25–37. doi: 10.1016/S0167-8140(17)31849-2 28056412

[14] Mudit C Patel KR Danish HH Lawson DH Khan MK . BRAF inhibitors and radiotherapy for melanoma brain metastases: potential advantages and disadvantages of combination therapy. Oncotargets Ther. (2016) 9:7149–59. doi: 10.2147/OTT.S119428, PMID: 28003758 PMC5161425

[15] Sambade MJ Peters EC Thomas NE Kaufmann WK Kimple RJ Shields JM . Melanoma cells show a heterogeneous range of sensitivity to ionizing radiation and are radiosensitized by inhibition of B-RAF with PLX-4032. Radiother Oncol. (2011) 98:394–9. doi: 10.1016/j.radonc.2010.12.017, PMID: 21295875 PMC3050997

[16] Hecht M Zimmer L Loquai C Weishaupt C Gutzmer R Schuster B . Radiosensitization by BRAF inhibitor therapy-mechanism and frequency of toxicity in melanoma patients. Ann Oncol. (2015) 26(6):1238–44. doi: 10.1093/annonc/mdv139, PMID: 25762352

[17] Yao Z Torres NM Tao A Gao Y Luo L Li Q . BRAF mutants evade ERK-dependent feedback by different mechanisms that determine their sensitivity to pharmacologic inhibition. Cancer Cell. (2015) 28:370–83. doi: 10.1016/j.ccell.2015.08.001, PMID: 26343582 PMC4894664

[18] Poulikakos PI Zhang C Bollag G Shokat KM Rosen N . RAF inhibitors transactivate RAF dimers and ERK signalling in cells with wild-type BRAF. Nature. (2010) 464:427–30. doi: 10.1038/nature08902, PMID: 20179705 PMC3178447

[19] Yi JS McCarthy KS Mazur K Kudlaty R Armstrong T Desai N . Sustained response to pan-BRAF inhibitor plixorafenib (FORE8394, PLX8394) in a young adult with neurodegenerative langerhans cell histiocytosis. JCO Precis Oncol. (2025) 9:e2500225. doi: 10.1200/PO-25-00225, PMID: 41066726 PMC12694702

[20] Holderfield M Deuker MM Mccormick F McMahon M . Targeting RAF kinases for cancer therapy: BRAF-mutated melanoma and beyond. Nat Rev Cancer. (2014) 14:455. doi: 10.1038/nrc3760, PMID: 24957944 PMC4250230

[21] Abu-Gheida I Chao S Murphy E Suh J Stevens GH Mohammadi AM . Targeted therapy after brain radiotherapy for BRAF-mutated melanoma with extensive ependymal disease with prolonged survival: case report and review of the literature. Front Oncol. (2019) 9:168. doi: 10.3389/fonc.2019.00168, PMID: 30972290 PMC6443873

[22] Kim JH Jenrow KA Brown SL . Mechanisms of radiation-induced normal tissue toxicity and implications for future clinical trials. Radiat Oncol J. (2014) 32:103–15. doi: 10.3857/roj.2014.32.3.103, PMID: 25324981 PMC4194292

[23] Ali FS Arevalo O Zorofchian S Patrizz A Riascos R Tandon N . Cerebral radiation necrosis: incidence, pathogenesis, diagnostic challenges, and future opportunities. Curr Oncol Rep. (2019) 21:66. doi: 10.1007/s11912-019-0818-y, PMID: 31218455

[24] Liu P Fu M Liu D Chao T Zhang J . Mechanisms of radiation-induced brain injury in mice based on bioinformatics analysis. Radiat Res. (2025) 203:321–32. doi: 10.1667/RADE-24-00204.1, PMID: 40133766

[25] Wu Q Fang Y Huang X Zheng F Ma S Zhang X . Role of orai3-mediated store-operated calcium entry in radiation-induced brain microvascular endothelial cell injury. Int J Mol Sci. (2023) 24(7):6818. doi: 10.3390/ijms24076818, PMID: 37047790 PMC10095176

[26] Wang Y Tian J Liu D Li T Mao Y Zhu C . Microglia in radiation-induced brain injury: Cellular and molecular mechanisms and therapeutic potential. CNS Neurosci Ther. (2024) 30:e14794. doi: 10.1111/cns.14794, PMID: 38867379 PMC11168970

[27] Zhao Z He X Gan L Xu D Zhang T Wang H . Investigation of the effects and mechanism of Total Glycosides of paeony against Radiation-Induced brain injury through network Pharmacology, molecular docking and experimental Verification. Int Immunopharmacol. (2025) 148:114178. doi: 10.1016/j.intimp.2025.114178, PMID: 39884083

[28] Borius P-Y Régis J Carpentier A Kalamarides M Valery CA Latorzeff I . Safety of radiosurgery concurrent with systemic therapy (chemotherapy, targeted therapy, and/or immunotherapy) in brain metastases: a systematic review. Cancer Metastasis Rev. (2021) 40:341–54. doi: 10.1007/s10555-020-09949-9, PMID: 33392851

[29] Verduin M Zindler JD Martinussen HMA Jansen RL Croes S Hendriks LE . Use of systemic therapy concurrent with cranial radiotherapy for cerebral metastases of solid tumors. Oncologist. (2017) 22:222–35. doi: 10.1634/theoncologist.2016-0117, PMID: 28167569 PMC5330699

[30] Baselet B Sonveaux P Baatout S Aerts A . Pathological effects of ionizing radiation: endothelial activation and dysfunction. Cell Mol Life ences CMLS. (2018) 76(4):699–728. doi: 10.1007/s00018-018-2956-z, PMID: 30377700 PMC6514067

[31] Andrews RN Metheny-Barlow LJ Peiffer AM Hanbury DB Tooze JA Bourland JD . Cerebrovascular remodeling and neuroinflammation is a late effect of radiation-induced brain injury in non-human primates. Radiat Res. (2017) 187:599–611. doi: 10.1667/RR14616.1, PMID: 28398880 PMC5508216

[32] Grant KG Gillespie Y Karamian A Lewin I Patel S Quigley A . Evolving treatment paradigms for melanoma brain metastases: A systematic review of current modalities. Clin Neurol Neurosurg. (2025) 257:109025. doi: 10.1016/j.clineuro.2025.109025, PMID: 40609368

[33] Dirven I Calliauw E Awada G Vounckx M Kessels JI Neyns B . Successful treatment of MAP2K1 mutant stage IV-M1d melanoma with trametinib plus low-dose dabrafenib: a case report. Front Med. (2024) 11:1436774. doi: 10.3389/fmed.2024.1436774, PMID: 39314226 PMC11418105

[34] Robb R Yang L Shen C Wolfe AR Webb A Zhang X . Inhibiting BRAF oncogene-mediated radioresistance effectively radiosensitizes BRAF(V600E)-mutant thyroid cancer cells by constraining DNA double-strand break repair. Clin Cancer Res. (2019) 25:4749–60. doi: 10.1158/1078-0432.CCR-18-3625, PMID: 31097454 PMC6677585

[35] Bhargava A Pelech S Woodard B Kerwin J Maherali N . Registered report: RAF inhibitors prime wild-type RAF to activate the MAPK pathway and enhance growth. Elife. (2016) 5:e09976. doi: 10.7554/eLife.09976, PMID: 26882073 PMC4769155

[36] Dong D Fu Y Chen F Zhang J Jia H Li J . Hyperoxia sensitizes hypoxic HeLa cells to ionizing radiation by downregulating HIF−1α and VEGF expression. Mol Med Rep. (2020) 23(1):62. doi: 10.3892/mmr.2020.11700, PMID: 33215223 PMC7706008

[37] Su F Bradley WD Wang Q Yang H Xu L Higgins B . Resistance to selective BRAF inhibition can be mediated by modest upstream pathway activation. Cancer Res. (2012) 72:969. doi: 10.1158/0008-5472.CAN-11-1875, PMID: 22205714

[38] Zhuang H Tao L Wang X Shi S Yuan Z Wang E . Tyrosine kinase inhibitor resistance increased the risk of cerebral radiation necrosis after stereotactic radiosurgery in brain metastases of non-small-cell lung cancer: A multi-institutional retrospective case-control study. Front Oncol. (2020) 10:12. doi: 10.3389/fonc.2020.00012, PMID: 32117704 PMC7026471

[39] Lan J Ren Y Liu Y Chen L Liu J . A bibliometric analysis of radiation-induced brain injury: a research of the literature from 1998 to 2023. Discov Oncol. (2024) 15:364. doi: 10.1007/s12672-024-01223-6, PMID: 39172266 PMC11341524

[40] Bromberger S Zadorozhna Y Ressler JM Holzner S Nawrocki A Zila N . Off-targets of BRAF inhibitors disrupt endothelial signaling and vascular barrier function. Life Sci Alliance. (2024) 7(8):e202402671. doi: 10.26508/lsa.202402671, PMID: 38839106 PMC11153892

[41] Li M Liu D Lee D Kapoor S Gibson-Corley KN Quinn TP . Enhancing the efficacy of melanocortin 1 receptor-targeted radiotherapy by pharmacologically upregulating the receptor in metastatic melanoma. Mol Pharm. (2019) 16:3904–15. doi: 10.1021/acs.molpharmaceut.9b00512, PMID: 31318566 PMC6765223

[42] Che J Sun Y Deng Y Zhang J . Blood-brain barrier disruption: a culprit of cognitive decline? Fluids Barriers CNS. (2024) 21:63. doi: 10.1186/s12987-024-00563-3, PMID: 39113115 PMC11305076

[43] Bland S Waldrup H . The role of stereotactic radiosurgery in combination with immunotherapy for metastatic melanoma following palliative surgery: a case report and review of the literature. World J Surg Oncol. (2025) 24(1):33. doi: 10.1186/s12957-025-04108-2, PMID: 41353395 PMC12797500

[44] Shao Y Wang Z Chen J Li J . Diffusion tensor imaging parameters for the early diagnosis of radiation-induced brain injury in patients with nasopharyngeal carcinoma: a meta-analysis. Int J Radiat Biol. (2023) 100:335–42. doi: 10.1080/09553002.2023.2280010, PMID: 37934054

[45] Perez WD Perez-Torres CJ . Neurocognitive and radiological changes after cranial radiation therapy in humans and rodents: a systematic review. Int J Radiat Biol. (2022) 99:119–37. doi: 10.1080/09553002.2022.2074167, PMID: 35511499

[46] Khan M Zheng T Zhao Z Arooj S Liao G . Efficacy of BRAF inhibitors in combination with stereotactic radiosurgery for the treatment of melanoma brain metastases: A systematic review and meta-analysis. Front Oncol. (2021) 10:586029. doi: 10.3389/fonc.2020.586029, PMID: 33692938 PMC7937920

[47] Geukes Foppen MH Boogerd W Blank CU van Thienen JV Haanen JB Brandsma D . Clinical and radiological response of BRAF inhibition and MEK inhibition in patients with brain metastases from BRAF-mutated melanoma. Melanoma Res. (2018) 28:126–33. doi: 10.1097/CMR.0000000000000429, PMID: 29356790

[48] Long GV Stroyakovskiy D Gogas H Levchenko E de Braud F Larkin J . Dabrafenib and trametinib versus dabrafenib and placebo for Val600 BRAF-mutant melanoma: a multicentre, double-blind, phase 3 randomised controlled trial. Lancet. (2015) 386:444–51. doi: 10.1016/S0140-6736(15)60898-4, PMID: 26037941

[49] Xue R Chen M Cai J Deng Z Pan D Liu X . Blood-brain barrier repair of bevacizumab and corticosteroid as prediction of clinical improvement and relapse risk in radiation-induced brain necrosis: A retrospective observational study. Front Oncol. (2021) 11:720417. doi: 10.3389/fonc.2021.720417, PMID: 34692494 PMC8526720

[50] Gupta K Perkerson RB Parsons TM Angom R Amerna D Burgess JD . Secretome from iPSC-derived MSCs exerts proangiogenic and immunosuppressive effects to alleviate radiation-induced vascular endothelial cell damage. Stem Cell Res Ther. (2024) 15:1–24. doi: 10.1186/s13287-024-03847-5, PMID: 39075600 PMC11287895

